# Next-Generation Cardiovascular Imaging in Precision Medicine: Integrating Functional Imaging, Artificial Intelligence, Biomarkers, and Personalized Risk Stratification

**DOI:** 10.3390/diagnostics16142230

**Published:** 2026-07-16

**Authors:** Carmine Siniscalchi, Manuela Basaglia, Vincenzo Russo, Pierpaolo Di Micco

**Affiliations:** 1 Internal Medicine Department, Parma University Hospital, 43125 Parma, Italy; csiniscalchi84@gmail.com (C.S.); mbasaglia80@gmail.com (M.B.); 2 Department of Translational Medical Sciences, University of Campania “Luigi Vanvitelli”-Monaldi Hospital, Piazzale Ettore Ruggeri, 80131 Naples, Italy; v.p.russo@libero.it; 3 Internal Medicine Ward, Santa Maria Delle Grazie Hospital, 80122 Naples, Italy

**Keywords:** cardiovascular imaging, vascular imaging, artificial intelligence, radiomics, echocardiography, cardiac magnetic resonance, photon-counting computed tomography, PET/CT, PET/MR, point-of-care ultrasound, venous thromboembolism, pulmonary embolism, precision medicine, risk stratification, biomarkers

## Abstract

Cardiovascular and vascular diseases remain major causes of morbidity and mortality worldwide, despite substantial advances in prevention, diagnosis, and treatment. In recent years, cardiovascular imaging has moved beyond the traditional assessment of anatomy and morphology toward a multidimensional evaluation of function, tissue composition, haemodynamics, inflammation, and individualized risk. This evolution has been driven by technological progress in echocardiography, cardiovascular magnetic resonance, computed tomography, nuclear imaging, intravascular imaging, and point-of-care ultrasound, together with the rapid development of artificial intelligence, radiomics, and predictive analytics. Advanced echocardiographic techniques, including contrast stress echocardiography and emerging methods for myocardial scar detection, may improve functional and prognostic assessment in patients with suspected or established coronary artery disease. Cardiac magnetic resonance, through tissue mapping, late gadolinium enhancement, and 4D flow imaging, provides unique information on myocardial fibrosis, perfusion, ventricular remodelling, and vascular haemodynamics. Computed tomography, particularly with the introduction of photon-counting technology, is expanding the non-invasive characterization of coronary plaques, vascular calcification, and thromboembolic disease. Hybrid imaging with PET/CT and PET/MR offers additional insight into vascular inflammation, myocardial metabolism, and active disease processes. At the same time, intravascular ultrasound, optical coherence tomography, and augmented-reality-supported imaging are refining interventional guidance, while point-of-care ultrasound is broadening access to rapid bedside cardiovascular and vascular assessment. The integration of imaging findings with circulating biomarkers, clinical scores, lipid profiles, coagulation parameters, and machine-learning models represents a promising strategy for personalized risk stratification, particularly in complex conditions such as coronary artery disease, venous thromboembolism, pulmonary embolism, and bleeding risk during antithrombotic therapy. This review summarizes current advances in cardiovascular imaging, discusses their translational implications, and highlights future directions for integrating imaging, artificial intelligence, and precision medicine into daily clinical practice.

## 1. Introduction

Cardiovascular and vascular diseases remain the leading causes of morbidity and mortality worldwide, accounting for nearly one-third of all deaths and representing a major challenge for healthcare systems despite substantial advances in prevention and treatment [1,2]. The growing burden of ischemic heart disease, heart failure, atrial fibrillation, peripheral arterial disease, aortic disease, and thromboembolic disorders is largely driven by population ageing, increasing prevalence of cardiometabolic risk factors, and improved survival of patients with chronic cardiovascular conditions [3]. Consequently, there is an increasing need for diagnostic tools capable not only of detecting disease but also of accurately stratifying risk, predicting outcomes, and guiding individualized therapeutic strategies.

Over the last two decades, cardiovascular imaging has undergone a profound transformation. Historically, imaging modalities were primarily used to assess anatomy and identify structural abnormalities. Today, technological innovations allow clinicians to investigate myocardial function, tissue composition, vascular inflammation, haemodynamics, plaque vulnerability, and disease activity with unprecedented precision [4]. As a result, cardiovascular imaging is progressively evolving from a diagnostic discipline into a central component of precision medicine.

The concept of personalized cardiovascular care relies on the integration of clinical information, laboratory biomarkers, imaging findings, genetic factors, and computational models to tailor management strategies according to the characteristics of individual patients [5]. Imaging techniques are particularly well suited to support this paradigm because they provide non-invasive access to pathophysiological mechanisms that were previously difficult or impossible to evaluate in routine clinical practice. Modern imaging modalities can identify myocardial fibrosis, quantify coronary plaque burden, assess microvascular dysfunction, detect vascular inflammation, characterize thrombotic disease, and evaluate biomechanical parameters associated with disease progression and clinical outcomes [6].

Among currently available modalities, echocardiography remains the most widely used cardiovascular imaging technique because of its accessibility, portability, safety profile, and cost-effectiveness. At the same time, recent advances in strain imaging, myocardial work analysis, contrast echocardiography, and stress imaging have considerably expanded its diagnostic and prognostic capabilities [7]. Similarly, cardiovascular magnetic resonance (CMR) has emerged as the reference standard for tissue characterization, enabling comprehensive assessment of myocardial fibrosis, inflammation, perfusion, and ventricular function through advanced techniques such as late gadolinium enhancement, T1/T2 mapping, and four-dimensional flow imaging [8].

Computed tomography (CT) has also experienced significant technological evolution. Coronary CT angiography now plays a pivotal role in the assessment of coronary artery disease, while newer developments such as photon-counting CT are opening new possibilities for plaque characterization and vascular imaging [9]. In parallel, molecular imaging modalities including positron emission tomography/computed tomography (PET/CT) and positron emission tomography/magnetic resonance (PET/MR) provide unique insights into biological processes such as inflammation, metabolism, and active atherosclerotic disease, facilitating a more comprehensive understanding of cardiovascular pathology [10].

Another major area of innovation concerns interventional and intravascular imaging. Technologies such as intravascular ultrasound (IVUS) and optical coherence tomography (OCT) have improved the characterization of coronary plaque morphology and procedural guidance during percutaneous interventions. Furthermore, advances in image fusion, augmented reality, and real-time computational analysis are creating new opportunities for precision-guided cardiovascular therapies [11].

Beyond highly specialized imaging platforms, point-of-care ultrasound (POCUS) is increasingly extending the reach of cardiovascular imaging to emergency departments, outpatient settings, and resource-limited environments. The ability to perform rapid bedside assessment of cardiac function, vascular abnormalities, and haemodynamic status has important implications for early diagnosis and clinical decision-making [12].

Current cardiovascular guidelines increasingly incorporate advanced imaging findings into risk stratification algorithms and therapeutic pathways. Nevertheless, several challenges remain. These include the need for standardization across imaging platforms, integration of multimodal datasets, optimization of cost-effectiveness, and incorporation of artificial intelligence (AI) into routine clinical workflows. Machine learning, radiomics, and automated image analysis are expected to play a central role in addressing these challenges and facilitating the transition toward truly personalized cardiovascular medicine.

The principal strengths, limitations, and emerging applications of contemporary cardiovascular imaging modalities are summarized in Table 1. In addition, the growing integration of imaging biomarkers with laboratory parameters, clinical variables, and AI-driven predictive models is outlined in Table 2. Representative applications of artificial intelligence across the major cardiovascular imaging modalities are summarized in Table 3.

The aim of this review is to provide a comprehensive overview of current advances in cardiovascular imaging, focusing on emerging technologies and their role in personalized risk stratification. Particular attention will be devoted to advanced echocardiography, cardiovascular magnetic resonance, computed tomography, molecular imaging, intravascular imaging, point-of-care ultrasound, and artificial intelligence, emphasizing how these innovations may contribute to the development of next-generation precision cardiovascular medicine.

Unlike previous reviews focused on individual imaging modalities, the present review integrates anatomical, functional, molecular, and intravascular imaging with artificial intelligence, digital health technologies, biomarkers, and precision medicine. Furthermore, it emphasizes future translational strategies aimed at integrating multimodal data for individualized cardiovascular risk stratification.

## 2. Advanced Echocardiography and Functional Imaging: From Conventional Assessment to Precision Risk Stratification

Echocardiography remains the cornerstone of cardiovascular imaging and continues to be the first-line diagnostic modality in most cardiovascular conditions. Its widespread availability, portability, absence of ionizing radiation, and relatively low cost have ensured its central role in clinical practice for several decades. However, the field has evolved substantially beyond conventional two-dimensional imaging and Doppler assessment. Contemporary echocardiography increasingly incorporates advanced quantitative techniques that provide detailed information regarding myocardial mechanics, ventricular performance, coronary microvascular function, myocardial perfusion, and cardiovascular risk, thereby contributing to more individualized patient management [13,14].

Conventional echocardiographic parameters such as left ventricular ejection fraction (LVEF), chamber dimensions, wall thickness, and valvular function remain fundamental components of cardiovascular assessment. Nevertheless, it has become increasingly evident that these measures alone may not adequately reflect myocardial health or predict future cardiovascular events. In particular, reliance on LVEF may underestimate early myocardial dysfunction, especially in patients with coronary artery disease, diabetes mellitus, hypertension, chemotherapy-related cardiotoxicity, and heart failure with preserved ejection fraction [15].

One of the most important advances in modern echocardiography has been the introduction of speckle-tracking echocardiography (STE), which enables quantitative assessment of myocardial deformation through strain analysis. Global longitudinal strain (GLS) has emerged as a highly sensitive marker of subclinical myocardial dysfunction and frequently identifies abnormalities before reductions in LVEF become apparent. Studies have demonstrated the prognostic significance of GLS in a broad range of cardiovascular diseases, including ischemic heart disease, cardiomyopathies, valvular disorders, and heart failure syndromes [16,17]. The incorporation of strain imaging into routine practice has therefore enhanced the ability of echocardiography to support early diagnosis and risk stratification.

More recently, myocardial work analysis has been proposed as an additional refinement of myocardial functional assessment. By integrating strain measurements with non-invasive estimates of left ventricular pressure, myocardial work indices provide a more comprehensive evaluation of myocardial performance while partially accounting for loading conditions. Emerging evidence suggests that these parameters may improve prognostic assessment and treatment monitoring in patients with heart failure, ischemic cardiomyopathy, and valvular heart disease [18].

Stress echocardiography represents another area of major innovation. Initially developed as a technique for detecting inducible myocardial ischemia through assessment of regional wall motion abnormalities, stress echocardiography has evolved into a multiparametric imaging platform capable of evaluating myocardial perfusion, coronary flow reserve, ventricular contractile reserve, pulmonary congestion, and microvascular dysfunction [19]. This broader physiological assessment aligns closely with the principles of precision medicine, as it allows identification of specific mechanisms contributing to symptoms and cardiovascular risk.

Contrast-enhanced stress echocardiography has further improved diagnostic performance by enabling visualization of myocardial perfusion and enhancing endocardial border delineation. The use of ultrasound contrast agents increases diagnostic accuracy, particularly in patients with suboptimal acoustic windows, and provides incremental prognostic information beyond conventional wall-motion analysis [20]. Furthermore, the absence of ionizing radiation makes contrast stress echocardiography particularly attractive in contemporary cardiovascular imaging pathways.

The prognostic value of advanced stress echocardiographic techniques has been demonstrated in several clinical studies. In a prospective investigation involving patients with suspected coronary artery disease, Gaibazzi and colleagues reported that contrast stress echocardiography provided robust prognostic information comparable to vasodilator stress single-photon emission computed tomography (SPECT) for the prediction of hard cardiac events (cardiac death, non-fatal myocardial infarction, and urgent coronary revascularization) [21]. These findings support the role of advanced echocardiographic approaches as effective radiation-free alternatives for functional risk assessment in appropriately selected patients.

Another rapidly expanding field is the non-invasive assessment of coronary microvascular dysfunction. Increasing evidence indicates that microvascular abnormalities contribute significantly to myocardial ischemia, heart failure, and adverse cardiovascular outcomes, even in the absence of obstructive coronary artery disease. Doppler-derived coronary flow reserve measurements obtained during stress echocardiography provide valuable information regarding microvascular function and have demonstrated important prognostic implications across multiple patient populations [22].

Beyond functional assessment, emerging echocardiographic technologies are increasingly capable of providing information regarding myocardial tissue characteristics. Although cardiovascular magnetic resonance remains the reference standard for scar detection and tissue characterization, novel ultrasound-based approaches have shown promising results in identifying myocardial fibrosis and scar burden. This area is of particular clinical relevance because myocardial fibrosis is strongly associated with ventricular arrhythmias, sudden cardiac death, and heart failure progression.

A notable example is pulse-cancellation echocardiography, an innovative technique developed to improve visualization of myocardial scar tissue. In a study of patients with previous myocardial infarction undergoing implantable cardioverter-defibrillator implantation for primary prevention, Gaibazzi and colleagues demonstrated that myocardial scar detected by pulse-cancellation echocardiography was independently associated with appropriate defibrillator interventions during follow-up [23]. These findings suggest that echocardiographic scar characterization may contribute additional prognostic information beyond conventional risk markers and may help refine arrhythmic risk stratification strategies.

Three-dimensional echocardiography (3DE) has also expanded the capabilities of ultrasound imaging. Compared with traditional two-dimensional approaches, 3DE allows more accurate quantification of ventricular volumes, ejection fraction, and valvular anatomy while reducing geometric assumptions. This technology has become increasingly important in structural heart disease and interventional cardiology, particularly for procedural planning and guidance during transcatheter valve interventions and left atrial appendage closure procedures [24].

The future of echocardiography will likely be shaped by the integration of artificial intelligence, automation, and advanced quantitative analysis. Machine-learning algorithms are already being applied to image acquisition, chamber segmentation, functional assessment, and automated disease classification. These developments may improve reproducibility, reduce interobserver variability, and facilitate broader implementation of advanced echocardiographic techniques in routine clinical practice. Moreover, AI-driven analysis may enable integration of imaging findings with clinical and laboratory data, generating individualized risk profiles that support personalized therapeutic decision-making.

Despite its excellent diagnostic performance, echocardiography is unlikely to become a population-wide screening tool because of operator dependency, variability in image acquisition and interpretation, and the limited availability of experienced operators in many healthcare settings. Its greatest value currently lies in targeted assessment of selected high-risk populations rather than universal cardiovascular screening.

Taken together, these innovations illustrate the remarkable evolution of echocardiography from a predominantly anatomical imaging technique into a sophisticated platform capable of functional assessment, tissue characterization, prognostic stratification, and precision cardiovascular management. The principal echocardiographic applications and emerging clinical implications are summarized in Table 1, while their integration with biomarkers and predictive algorithms is presented in Table 2.

## 3. Cardiovascular Magnetic Resonance: Comprehensive Tissue Characterization and Functional Assessment

Cardiovascular magnetic resonance (CMR) has emerged as one of the most comprehensive imaging modalities in modern cardiovascular medicine. Owing to its high spatial resolution, excellent reproducibility, multiparametric capabilities, and absence of ionizing radiation, CMR is increasingly recognized as the reference standard for the assessment of cardiac volumes, ventricular function, myocardial tissue characterization, and complex cardiovascular anatomy [25]. Unlike other imaging techniques that primarily focus on anatomy or function, CMR provides simultaneous evaluation of morphology, perfusion, fibrosis, inflammation, viability, and haemodynamics, making it particularly well suited for precision medicine applications.

One of the most established clinical applications of CMR is the quantification of ventricular volumes and systolic function. The high accuracy and reproducibility of CMR measurements have made this modality the gold standard for assessing both left and right ventricular performance [26]. This capability is especially valuable in patients with congenital heart disease, pulmonary hypertension, arrhythmogenic cardiomyopathy, and complex structural abnormalities, where echocardiographic evaluation may be limited by acoustic windows or geometric assumptions.

Perhaps the most important contribution of CMR to contemporary cardiovascular imaging is its unparalleled ability to characterize myocardial tissue. The introduction of late gadolinium enhancement (LGE) imaging revolutionized the non-invasive evaluation of myocardial fibrosis and scar tissue. LGE enables direct visualization of replacement fibrosis and provides important diagnostic and prognostic information across a broad spectrum of cardiovascular diseases [27]. Distinct enhancement patterns allow differentiation between ischemic and non-ischemic myocardial injury, facilitating more accurate diagnosis and disease classification.

In patients with ischemic heart disease, the extent and transmurality of myocardial scar identified by LGE correlate closely with the likelihood of functional recovery following coronary revascularization [28]. Furthermore, studies have demonstrated that myocardial scar burden is strongly associated with ventricular arrhythmias, sudden cardiac death, and long-term cardiovascular outcomes, often independently of left ventricular ejection fraction [29]. Consequently, scar quantification has become an important component of contemporary risk stratification strategies.

Beyond focal fibrosis, newer parametric imaging techniques have substantially expanded the diagnostic capabilities of CMR. Native T1 mapping, T2 mapping, and extracellular volume (ECV) quantification allow detection of diffuse myocardial abnormalities that may not be visible using conventional LGE imaging [30]. These quantitative biomarkers provide insights into myocardial inflammation, edema, diffuse interstitial fibrosis, and infiltrative processes, enabling earlier diagnosis and more precise disease characterization.

The clinical impact of mapping techniques has been particularly evident in inflammatory and infiltrative cardiomyopathies. In myocarditis, contemporary diagnostic criteria increasingly incorporate T1- and T2-based imaging biomarkers because of their ability to identify myocardial inflammation and tissue injury non-invasively [31]. Similarly, CMR has transformed the evaluation of cardiac amyloidosis, Fabry disease, and other infiltrative disorders, providing characteristic imaging signatures that may facilitate diagnosis without the need for invasive biopsy procedures.

Stress perfusion CMR represents another major advance in cardiovascular imaging. Through the assessment of myocardial blood flow during pharmacological stress, this technique enables detection of inducible ischemia with excellent diagnostic accuracy. Several large multicentre studies have demonstrated that stress perfusion CMR performs favorably compared with other non-invasive functional imaging modalities for identifying haemodynamically significant coronary artery disease [32]. Importantly, negative stress CMR findings are associated with excellent long-term prognosis, further supporting its value in clinical decision-making.

Stress CMR is currently considered one of the reference standards for non-invasive functional assessment of myocardial ischemia because of its excellent diagnostic accuracy and absence of ionizing radiation. However, its use remains limited by cost, scanner availability, examination time, and the need for specialized personnel and emergency cardiovascular support during pharmacological stress testing.

The role of CMR extends beyond myocardial disease and increasingly encompasses vascular and haemodynamic assessment. Four-dimensional flow magnetic resonance imaging (4D-flow MRI) has emerged as one of the most innovative developments in the field. This technique enables comprehensive visualization and quantification of blood flow patterns throughout the cardiovascular system, providing information regarding wall shear stress, flow turbulence, vortex formation, and energy loss [33]. Such parameters offer unique insights into disease mechanisms that cannot be assessed using conventional imaging modalities.

Clinical applications of 4D-flow MRI continue to expand rapidly. Abnormal haemodynamic patterns have been associated with the development and progression of aortic aneurysms, bicuspid aortic valve disease, valvular heart disorders, and congenital cardiovascular abnormalities [34]. In addition, haemodynamic biomarkers derived from 4D-flow imaging may contribute to personalized surveillance strategies by identifying patients at increased risk of disease progression before structural changes become clinically apparent.

Recent advances in computational analysis and artificial intelligence are expected to further enhance the role of CMR in precision cardiovascular medicine. Machine-learning algorithms are increasingly used for automated segmentation, image reconstruction, tissue characterization, and prognostic modelling. These approaches may improve workflow efficiency while simultaneously extracting quantitative information beyond the capabilities of conventional visual interpretation [35]. Integration of CMR-derived biomarkers with clinical variables, laboratory parameters, and other imaging modalities may ultimately facilitate highly individualized risk prediction and therapeutic decision-making.

The major strengths and clinical applications of CMR are summarized in Table 1. Furthermore, several CMR-derived biomarkers—including myocardial scar burden, extracellular volume expansion, mapping abnormalities, and haemodynamic parameters—are increasingly incorporated into multimodal prediction models that combine imaging findings with laboratory biomarkers and artificial intelligence, as illustrated in Table 2.

Overall, CMR represents one of the clearest examples of the transition from conventional imaging toward precision cardiovascular medicine. By providing detailed information on myocardial structure, tissue composition, function, and haemodynamics within a single examination, CMR has become an indispensable component of contemporary cardiovascular risk stratification and personalized patient management.

## 4. Computed Tomography and Photon-Counting CT: From Coronary Anatomy to Plaque Biology

Computed tomography (CT) has become one of the most influential imaging modalities in contemporary cardiovascular medicine. Continuous improvements in scanner technology, image reconstruction algorithms, temporal resolution, and radiation dose reduction have transformed CT from a technique primarily used for anatomical assessment into a comprehensive tool capable of supporting diagnosis, prognostic stratification, procedural planning, and personalized risk prediction [36]. Among the most important developments has been the widespread adoption of coronary computed tomography angiography (CCTA), which now occupies a central position in the evaluation of patients with suspected coronary artery disease (CAD).

Beyond anatomical coronary assessment, stress CT myocardial perfusion imaging has emerged as an important functional imaging modality capable of identifying myocardial ischemia with high diagnostic accuracy. Combined anatomical and functional CT assessment may improve patient selection for invasive angiography, although widespread implementation remains limited by scanner availability, radiation exposure, contrast administration, and the need for dedicated expertise and appropriate emergency support.

The diagnostic performance of CCTA has been extensively validated in large multicentre studies and randomized clinical trials. Owing to its high sensitivity and excellent negative predictive value, CCTA allows reliable exclusion of significant coronary stenosis and has become a recommended first-line imaging modality in many patients presenting with stable chest pain [37]. Contemporary guidelines increasingly emphasize the role of CT-based anatomical assessment within diagnostic algorithms, reflecting growing evidence that early identification of coronary atherosclerosis may improve risk stratification and preventive management [38].

One of the major advantages of CCTA is its ability to evaluate not only luminal narrowing but also the characteristics of atherosclerotic plaques. This capability has substantially improved understanding of coronary artery disease as a complex biological process rather than a simple mechanical obstruction. Indeed, many acute coronary syndromes originate from lesions that are only mildly obstructive but exhibit morphological features associated with plaque vulnerability [39].

Several CT-derived plaque characteristics have been associated with increased cardiovascular risk. Positive remodelling, low-attenuation plaque, spotty calcification, and the napkin-ring sign have all been linked to plaque instability and future cardiovascular events [40]. Nevertheless, anatomical plaque characterization should not be considered synonymous with functionally significant ischemic heart disease. Anatomical stenosis severity and myocardial ischemia frequently diverge, particularly in intermediate coronary lesions. Therefore, anatomical plaque assessment should ideally be integrated with functional imaging modalities when therapeutic decisions are considered. These findings have stimulated growing interest in plaque phenotyping and have contributed to a paradigm shift from stenosis-centred evaluation toward comprehensive assessment of atherosclerotic disease activity.

Another important advance has been the development of imaging biomarkers capable of identifying vascular inflammation. Perivascular adipose tissue is increasingly recognized as a dynamic biological compartment that reflects inflammatory processes occurring within the adjacent vascular wall. Changes in CT attenuation of perivascular fat have been associated with coronary inflammation and adverse cardiovascular outcomes, suggesting that CT may provide indirect information regarding disease activity beyond conventional anatomical assessment [41]. Such biomarkers represent an important step toward integrating structural and biological information within a single imaging examination.

Coronary artery calcium (CAC) scoring remains another highly valuable application of CT imaging. Studies have demonstrated that CAC burden is one of the strongest predictors of future cardiovascular events in asymptomatic individuals [42]. The ability of CAC scoring to improve risk classification beyond traditional clinical models has led to its incorporation into several international prevention guidelines. Conversely, the absence of coronary calcification identifies individuals with a particularly favorable prognosis and may support more individualized preventive strategies [43].

The clinical applications of CT extend well beyond coronary artery disease. CT angiography plays a fundamental role in the evaluation of thoracic aortic disease, peripheral arterial disease, congenital vascular abnormalities, and structural heart disorders. In addition, CT has become indispensable for procedural planning in transcatheter interventions such as transcatheter aortic valve replacement, left atrial appendage occlusion, and complex electrophysiological procedures [44]. Three-dimensional reconstructions provide detailed anatomical information that may improve procedural accuracy and reduce complications.

CT imaging also represents the cornerstone of contemporary pulmonary embolism diagnosis. Computed tomography pulmonary angiography (CTPA) has largely replaced older diagnostic approaches because of its high diagnostic accuracy, rapid acquisition, and widespread availability [45]. Importantly, modern CTPA provides prognostic information beyond the mere detection of thrombi. Imaging markers such as right ventricular enlargement, interventricular septal deviation, and clot burden have been associated with short-term outcomes and may contribute to individualized risk stratification in patients with acute pulmonary embolism [46].

Perhaps the most exciting recent innovation in CT technology is the development of photon-counting computed tomography (PCCT). Unlike conventional energy-integrating detector systems, photon-counting detectors directly register individual X-ray photons and measure their energy. This approach offers several theoretical and practical advantages, including improved spatial resolution, enhanced contrast-to-noise ratio, reduced image artefacts, and lower radiation exposure [47].

The potential implications of PCCT for cardiovascular imaging are substantial. Improved spatial resolution may facilitate more accurate visualization of coronary plaques, small vascular structures, heavily calcified lesions, and intracoronary stents. Furthermore, spectral information generated by photon-counting detectors allows more precise differentiation of tissue components, potentially improving characterization of lipid-rich plaques, fibrotic tissue, calcifications, and thrombotic material [48].

Early clinical investigations have demonstrated encouraging results regarding coronary plaque assessment and quantitative tissue characterization. By reducing blooming artefacts associated with calcification, PCCT may improve diagnostic performance in patient populations that have traditionally represented a challenge for conventional CT systems [49]. Moreover, the ability to obtain high-quality images at lower radiation doses aligns closely with current efforts to optimize patient safety while maintaining diagnostic accuracy.

An additional area of rapid development involves radiomics and advanced image analysis. Radiomics enables extraction of large numbers of quantitative imaging features related to texture, shape, spatial distribution, and signal intensity. These features may capture biologically relevant information that is not readily identifiable through conventional visual interpretation [50]. Studies have suggested that CT-based radiomics may improve prediction of plaque vulnerability, cardiovascular events, and treatment response, although further validation is required before widespread clinical implementation.

Artificial intelligence is expected to further enhance the role of CT in precision cardiovascular medicine. Machine-learning algorithms are increasingly being used for automated coronary segmentation, plaque characterization, stenosis quantification, and risk prediction. By integrating imaging findings with clinical variables and laboratory biomarkers, AI-driven models may facilitate more individualized assessment of cardiovascular risk and support therapeutic decision-making [51].

Taken together, these advances illustrate the remarkable evolution of CT imaging from a purely anatomical modality into a sophisticated platform capable of evaluating plaque biology, vascular inflammation, haemodynamic consequences, and future cardiovascular risk. The principal applications, strengths, and limitations of conventional CT and emerging photon-counting CT technologies are summarized in Table 1. Furthermore, CT-derived biomarkers such as coronary calcium burden, high-risk plaque features, and inflammatory imaging signatures are increasingly incorporated into integrated risk prediction models that combine imaging findings, laboratory biomarkers, and artificial intelligence tools, as shown in Table 2.

As technology continues to evolve, CT is expected to play an increasingly important role in the transition toward precision cardiovascular medicine, providing clinicians with detailed anatomical, functional, and biological information that may improve patient selection, personalize therapeutic strategies, and ultimately enhance cardiovascular outcomes.

## 5. Molecular and Hybrid Imaging: PET/CT and PET/MR for Vascular Inflammation and Myocardial Metabolism

The growing recognition that cardiovascular diseases are driven not only by structural abnormalities but also by complex biological and inflammatory processes has stimulated increasing interest in molecular imaging. Unlike conventional imaging modalities, which primarily assess anatomy and function, molecular imaging allows visualization of cellular activity, metabolic pathways, inflammatory responses, and disease activity in vivo. Among currently available techniques, positron emission tomography (PET), particularly when integrated with computed tomography (PET/CT) or magnetic resonance imaging (PET/MR), represents the most established molecular imaging platform in cardiovascular medicine [47].

Atherosclerosis is now widely recognized as a chronic inflammatory disease involving interactions among endothelial dysfunction, lipid accumulation, immune activation, and vascular remodeling. Although anatomical imaging can identify plaque burden and stenosis severity, it provides limited information regarding biological activity within the vessel wall. Molecular imaging addresses this limitation by enabling direct or indirect assessment of inflammatory processes that contribute to plaque progression and destabilization [48].

The most commonly used radiotracer in cardiovascular PET imaging is fluorine-18 fluorodeoxyglucose (^18^F-FDG), which accumulates in metabolically active inflammatory cells, particularly activated macrophages. Increased vascular FDG uptake has been associated with inflammatory activity within atherosclerotic plaques and has been linked to future cardiovascular events [49]. Several studies have demonstrated correlations between FDG uptake and histological markers of inflammation, supporting the concept that PET imaging may identify biologically active lesions before structural complications become clinically apparent [50].

Beyond coronary atherosclerosis, FDG-PET has proven valuable in the assessment of inflammatory cardiovascular disorders. In large-vessel vasculitis, PET imaging facilitates early diagnosis, evaluation of disease extent, and monitoring of treatment response. Similarly, FDG-PET has become an important component of the diagnostic work-up for cardiac sarcoidosis, prosthetic valve endocarditis, cardiac implantable electronic device infections, and selected cases of myocarditis [51]. The ability to identify active inflammation may have important implications for therapeutic decision-making and long-term patient management.

Despite its utility, FDG imaging presents several limitations, including physiological myocardial glucose uptake that may complicate interpretation. Consequently, substantial research efforts have focused on the development of alternative radiotracers capable of more specifically targeting pathological processes involved in cardiovascular disease. Among these, fluorine-18 sodium fluoride (^18^F-NaF) has emerged as one of the most promising agents. Unlike FDG, NaF binds to areas of active microcalcification and may identify plaques undergoing active remodeling and progression [52].

Several investigations have demonstrated associations between coronary NaF uptake and high-risk plaque features identified by intravascular imaging and CT angiography. Importantly, NaF uptake appears to reflect ongoing biological activity rather than cumulative plaque burden, suggesting a potential role in identifying vulnerable plaques before the occurrence of acute cardiovascular events [53]. Although further validation is required, these findings highlight the growing potential of molecular imaging to contribute to individualized cardiovascular risk assessment.

Hybrid PET/CT imaging combines molecular and anatomical information within a single examination, providing complementary insights into both plaque morphology and biological activity. This integration enables more accurate localization of inflammatory signals and facilitates comprehensive evaluation of cardiovascular disease. By simultaneously assessing structural abnormalities and underlying pathophysiological mechanisms, PET/CT may improve identification of high-risk patients and support more personalized therapeutic strategies [54].

Molecular imaging also plays a crucial role in the evaluation of myocardial viability and metabolism. In patients with ischemic cardiomyopathy, PET can distinguish viable but dysfunctional myocardium from irreversibly scarred tissue. This information remains clinically relevant when considering coronary revascularization in patients with severe left ventricular dysfunction, as the presence of viable myocardium may predict functional recovery and improved outcomes following intervention [55].

PET myocardial perfusion imaging also represents one of the most accurate non-invasive methods for the diagnosis of ischemic heart disease, allowing absolute quantification of myocardial blood flow and coronary flow reserve. Despite its excellent diagnostic performance, widespread clinical use is limited by high costs, restricted availability, and the requirement for highly specialized multidisciplinary teams.

The development of hybrid PET/MR systems represents a further step toward comprehensive cardiovascular phenotyping. PET/MR combines the molecular sensitivity of PET with the superior tissue characterization capabilities of cardiovascular magnetic resonance. Consequently, clinicians can simultaneously assess myocardial inflammation, fibrosis, scar burden, ventricular function, perfusion abnormalities, and metabolic activity within a single examination [56].

This integrated approach appears particularly promising in inflammatory cardiomyopathies, infiltrative diseases, and complex vascular disorders. The combination of CMR-derived tissue characterization and PET-derived metabolic information may provide a more complete understanding of disease activity than either modality alone. Although PET/MR remains largely confined to specialized centres because of technical complexity and cost, ongoing technological improvements may facilitate broader clinical adoption in the future.

Recent advances in quantitative image analysis and artificial intelligence are expected to further expand the clinical applications of molecular imaging. Machine-learning algorithms can integrate molecular imaging findings with anatomical imaging features, laboratory biomarkers, and clinical variables to generate personalized risk profiles and predictive models. Such approaches may improve identification of patients at elevated risk of disease progression and support more targeted therapeutic interventions.

Despite considerable promise, several challenges currently limit widespread implementation of PET-based cardiovascular imaging. These include high costs, limited availability, radiation exposure, lack of standardization, and the need for specialized expertise. Furthermore, large prospective studies are still required to determine whether molecular imaging-guided management strategies consistently translate into improved clinical outcomes.

Nevertheless, molecular and hybrid imaging represent one of the most exciting frontiers in contemporary cardiovascular medicine. By enabling visualization of disease activity at the cellular and molecular levels, PET/CT and PET/MR move beyond traditional anatomical assessment and contribute directly to the goals of precision medicine. The principal applications of these modalities are summarized in Table 1, while their integration with biomarkers, clinical variables, and artificial intelligence-driven prediction models is outlined in Table 2.

## 6. Intravascular Imaging and Image-Guided Interventions: IVUS, OCT and Emerging Technologies

Although intravascular imaging is not suitable for population-based cardiovascular screening, it plays a fundamental role in individualized risk assessment and optimization of coronary interventions in selected high-risk patients. Therefore, it remains an essential component of contemporary precision cardiovascular medicine. By allowing direct visualization of the vessel wall and plaque architecture from within the artery, intravascular imaging modalities have significantly improved understanding of coronary artery disease pathophysiology and have enhanced the precision of percutaneous coronary interventions (PCI). Among available technologies, intravascular ultrasound (IVUS) and optical coherence tomography (OCT) have become the most widely adopted modalities in contemporary interventional cardiology [57].

IVUS was the first intravascular imaging technique to gain widespread clinical acceptance. By using high-frequency ultrasound transducers mounted on intracoronary catheters, IVUS provides cross-sectional images of the coronary vessel wall, allowing accurate assessment of plaque burden, vessel remodeling, lumen dimensions, and stent expansion [58]. Compared with conventional angiography, which only visualizes the vessel lumen, IVUS enables a more comprehensive evaluation of coronary atherosclerosis and often reveals substantial plaque accumulation even in angiographically mild lesions.

Studies have demonstrated the clinical value of IVUS-guided PCI. Optimization of stent sizing, expansion, and apposition through IVUS guidance has been associated with reductions in stent thrombosis, target lesion failure, and repeat revascularization procedures, particularly in complex coronary lesions [59]. Consequently, contemporary clinical guidelines increasingly recommend IVUS guidance in selected high-risk scenarios, including left main disease, long lesions, chronic total occlusions, and heavily calcified vessels.

Optical coherence tomography represents a more recent technological advancement that provides significantly higher spatial resolution than IVUS. By using near-infrared light rather than ultrasound waves, OCT generates extremely detailed images of the vessel wall and plaque microstructure [60]. This superior resolution allows visualization of features that are often beyond the capabilities of IVUS, including thin fibrous caps, macrophage accumulation, microcalcifications, plaque erosions, and small thrombi.

The ability of OCT to characterize plaque morphology has substantially improved understanding of the mechanisms underlying acute coronary syndromes. In particular, OCT studies have demonstrated that plaque rupture, plaque erosion, and calcified nodules represent distinct pathological substrates associated with different clinical presentations and potentially different therapeutic approaches [61]. Such observations support the growing concept that individualized treatment strategies may be guided by detailed characterization of plaque phenotype rather than solely by angiographic appearance.

Beyond plaque assessment, OCT has become an important tool for procedural optimization during PCI. High-resolution visualization of stent deployment enables identification of underexpansion, malapposition, edge dissections, tissue prolapse, and residual thrombus. Several randomized studies have demonstrated that OCT-guided PCI improves procedural results and may contribute to better long-term clinical outcomes compared with angiography-guided interventions alone [62].

The integration of intravascular imaging into routine clinical practice has also stimulated the development of multimodality imaging strategies. Increasingly, anatomical information obtained from CT angiography is combined with intravascular imaging findings to improve lesion characterization and procedural planning. Such approaches facilitate more comprehensive assessment of coronary disease and support personalized treatment decisions.

Emerging technologies are expected to further expand the capabilities of intravascular imaging. Near-infrared spectroscopy (NIRS), hybrid IVUS-NIRS systems, intravascular photoacoustic imaging, and molecular imaging catheters are currently being investigated as tools capable of identifying lipid-rich plaques, inflammatory activity, and other biological features associated with plaque vulnerability [63]. Although many of these technologies remain investigational, they illustrate the continuing evolution of intravascular imaging from purely structural assessment toward biological characterization of coronary disease.

Artificial intelligence is also beginning to influence the field of image-guided interventions. Automated plaque characterization, lesion segmentation, calcium quantification, and procedural planning algorithms are being developed to improve workflow efficiency and reduce operator variability. Furthermore, integration of intravascular imaging findings with clinical, laboratory, and non-invasive imaging data may facilitate more sophisticated prediction models capable of identifying patients at highest risk of future cardiovascular events [64].

Another promising area involves the incorporation of advanced visualization technologies into interventional procedures. Image fusion, three-dimensional reconstruction, virtual reality, and augmented reality platforms may improve procedural guidance and operator performance by integrating information from multiple imaging modalities in real time. Although these technologies remain at an early stage of clinical implementation, they represent important components of future precision-guided cardiovascular interventions.

Taken together, IVUS, OCT, and emerging intravascular imaging technologies have significantly enhanced the understanding and treatment of coronary artery disease. By providing detailed information regarding plaque morphology, vessel wall characteristics, and procedural outcomes, these techniques contribute directly to individualized patient management and precision interventional cardiology. Their principal applications, advantages, and limitations are summarized in Table 1, while their integration within broader multimodal risk stratification frameworks is illustrated in Table 2.

## 7. Point-of-Care Ultrasound: Expanding Cardiovascular Imaging Beyond the Echocardiography Laboratory

Point-of-care ultrasound (POCUS) has emerged as one of the most rapidly expanding applications of medical imaging and is increasingly reshaping cardiovascular and vascular diagnostics. Unlike conventional imaging pathways, which often require dedicated equipment, specialized laboratories, and delayed interpretation, POCUS enables clinicians to obtain real-time diagnostic information directly at the bedside. This capability has important implications for emergency medicine, critical care, internal medicine, cardiology, and outpatient practice, where rapid decision-making is frequently required [65].

The widespread adoption of portable and handheld ultrasound devices has significantly increased accessibility to cardiovascular imaging. Technological improvements in image quality, miniaturization, battery performance, and wireless connectivity have transformed ultrasound from a highly specialized diagnostic tool into an extension of the physical examination. Consequently, POCUS is increasingly described as the “visual stethoscope” of modern medicine, providing immediate anatomical and functional information that complements traditional clinical assessment [66].

One of the most important applications of cardiovascular POCUS is the rapid evaluation of patients presenting with acute dyspnea, chest pain, syncope, hypotension, or signs of circulatory instability. Bedside assessment of ventricular function, chamber size, pericardial effusion, volume status, and gross valvular abnormalities can often be performed within minutes and may substantially influence diagnostic and therapeutic decisions. In emergency and critical care settings, such rapid assessment may accelerate diagnosis while reducing the need for more time-consuming or resource-intensive investigations [67].

POCUS has also become an important tool in the management of heart failure. Evaluation of left ventricular systolic function, inferior vena cava dynamics, pulmonary congestion, and pleural effusions can provide valuable information regarding haemodynamic status and treatment response. Lung ultrasound, in particular, has demonstrated high sensitivity for detecting pulmonary congestion through the identification of B-lines and is increasingly incorporated into heart failure management pathways [68].

Another area in which POCUS has shown considerable clinical utility is vascular imaging. Compression ultrasonography performed at the bedside allows rapid assessment for deep vein thrombosis (DVT), particularly in emergency departments and acute care settings. Several studies have demonstrated that focused ultrasound protocols performed by appropriately trained clinicians can achieve high diagnostic accuracy for proximal lower-limb DVT, facilitating earlier diagnosis and treatment initiation [69]. Given the clinical importance of venous thromboembolism and its associated morbidity and mortality, bedside vascular ultrasound represents a valuable component of modern patient care.

The role of POCUS extends beyond diagnosis and increasingly includes procedural guidance. Ultrasound-guided vascular access, pericardiocentesis, thoracentesis, and other bedside procedures have been associated with improved success rates and reduced complication rates compared with landmark-based approaches. Consequently, ultrasound guidance is now considered standard practice for many invasive procedures across multiple specialties.

Recent technological developments are further expanding the capabilities of POCUS. Handheld devices equipped with artificial intelligence-assisted image acquisition and automated interpretation algorithms are becoming increasingly available. These systems may facilitate broader adoption among non-expert users and improve diagnostic consistency in resource-limited environments. Furthermore, integration with telemedicine platforms allows remote supervision and consultation, creating new opportunities for cardiovascular care in underserved regions [70].

Despite its many advantages, POCUS should not be considered a replacement for comprehensive cardiovascular imaging. Image quality may be limited, diagnostic accuracy depends heavily on operator training and experience, and detailed tissue characterization remains beyond the capabilities of most portable devices. Rather, POCUS should be viewed as a complementary tool that extends the reach of cardiovascular imaging and facilitates rapid clinical decision-making.

Future developments are likely to focus on improved image quality, cloud-based data sharing, automated interpretation, and integration with artificial intelligence-driven decision support systems. Such innovations may further enhance the role of POCUS within precision medicine frameworks by enabling earlier diagnosis, more efficient triage, and personalized monitoring strategies.

The major cardiovascular and vascular applications of POCUS are summarized in Table 1. In addition, its integration with digital health technologies, artificial intelligence platforms, and predictive clinical models is outlined in Table 2, highlighting the growing role of bedside imaging within next-generation cardiovascular care.

## 8. Artificial Intelligence, Mobile Health, Biomarkers and Precision Cardiovascular Medicine

The rapid expansion of cardiovascular imaging technologies has generated unprecedented amounts of clinical and imaging data. While advanced imaging modalities provide increasingly detailed information regarding anatomy, function, tissue composition, inflammation, and haemodynamics, the interpretation and integration of these complex datasets remain significant challenges. In this context, artificial intelligence (AI), machine learning, radiomics, digital health technologies, and biomarker-based approaches are emerging as fundamental components of next-generation cardiovascular medicine [71].

Deep-learning algorithms have demonstrated remarkable performance in automated image acquisition, segmentation, chamber quantification, plaque characterization, tissue classification, and diagnostic interpretation across echocardiography, CMR, CT, and nuclear imaging [72]. By reducing interobserver variability and improving efficiency, AI may facilitate broader implementation of advanced imaging techniques while maintaining high diagnostic standards.

AI applications differ substantially across cardiovascular imaging modalities. In echocardiography, AI is currently used for automated image acquisition guidance, view classification, left ventricular segmentation, chamber quantification, strain analysis, and automated reporting. In CMR, AI supports accelerated image reconstruction, tissue segmentation, automated ventricular volumetry, T1/T2 mapping analysis, and scar quantification. In CT imaging, AI enables coronary artery segmentation, plaque characterization, calcium scoring, stenosis quantification, radiomic feature extraction, and CT-derived fractional flow reserve estimation. In PET imaging, AI contributes to image denoising, motion correction, quantitative tracer analysis, and multimodal image fusion. These applications span the entire imaging workflow, from acquisition to quantitative interpretation and clinical decision support, although their degree of clinical maturity differs considerably.

Increasingly, AI is being integrated upstream within the imaging workflow rather than functioning solely as a post-processing tool. Modern algorithms optimize scan acquisition, reduce operator dependency during image acquisition, accelerate image reconstruction, improve image quality through denoising and artifact reduction, automatically assess image quality, prioritize urgent examinations for reporting, and streamline workflow management. These developments may shorten acquisition time, improve reproducibility, reduce reporting delays, and increase the reliability of downstream quantitative measurements.

Beyond image interpretation, machine-learning models are increasingly being used for prognostic assessment and clinical decision support. Unlike traditional statistical models, machine-learning algorithms can simultaneously analyze large numbers of variables and identify complex nonlinear relationships among imaging findings, laboratory biomarkers, and clinical characteristics. Several studies have demonstrated that AI-based prediction models may outperform conventional risk scores in forecasting cardiovascular events, disease progression, and treatment response [73].

Radiomics represents another rapidly developing field within cardiovascular imaging. By extracting large numbers of quantitative imaging features from standard medical images, radiomics enables characterization of tissue heterogeneity and disease phenotypes beyond what is visible to the human eye [74]. Radiomic signatures derived from CT, CMR, PET, and echocardiographic datasets have shown promising results for identifying vulnerable plaques, predicting adverse cardiovascular outcomes, and improving diagnostic classification. Although further standardization and validation are needed, radiomics may become an important component of future precision medicine strategies.

More recently, generalist and foundation AI models have emerged as a promising direction for cardiovascular imaging. Unlike task-specific algorithms, these models are trained on large multimodal datasets incorporating echocardiography, CT, CMR, PET, clinical information, and text, allowing transfer learning across multiple imaging tasks. Such architectures may facilitate scalable deployment across heterogeneous healthcare environments while improving generalizability and reducing the need for task-specific retraining [75].

The emergence of mobile health technologies has further expanded opportunities for personalized cardiovascular care. Wearable devices, smartphone-based monitoring systems, remote sensors, and telemedicine platforms now allow continuous collection of physiological and behavioural data outside traditional healthcare settings [76]. Such technologies facilitate early detection of clinical deterioration, improve patient engagement, and support longitudinal disease monitoring.

Recent advances in personalized cardiology also include the integration of artificial intelligence with digital electrocardiography. AI-assisted interpretation of conventional 12-lead ECGs and wearable single-lead ECG devices has demonstrated promising performance for the detection of atrial fibrillation, silent left ventricular dysfunction, hypertrophic cardiomyopathy, electrolyte abnormalities, and individualized cardiovascular risk prediction. Owing to their low cost, wide availability, and scalability, digital ECG technologies may become one of the most practical tools for population-based cardiovascular screening when integrated with machine-learning algorithms.

Additional non-invasive approaches are also emerging within precision cardiology. Assessment of arterial stiffness using pulse wave velocity, augmentation index, and central blood pressure provides important information regarding vascular aging and cardiovascular risk beyond traditional risk factors. Furthermore, volatilomic analysis, based on the characterization of volatile organic compounds in exhaled breath, represents an innovative biomarker platform currently being investigated for cardiovascular disease detection, metabolic profiling, and individualized risk stratification. Future integration of these technologies with multimodal imaging and AI-based predictive models may further improve personalized cardiovascular care.

An illustrative example of this approach is the Heart Sentinel™ application, a smartphone-based system developed to detect cardiac arrest during outdoor physical activity and automatically activate emergency alert procedures. Field testing and ventricular fibrillation simulation studies demonstrated the feasibility of this innovative approach, highlighting the potential role of mobile health technologies in improving emergency cardiovascular response and patient safety [77]. Although not an imaging modality per se, the integration of digital monitoring platforms with advanced cardiovascular imaging and predictive algorithms exemplifies the broader evolution toward connected and personalized healthcare systems.

A key principle of precision medicine is the integration of imaging findings with laboratory biomarkers. Increasing evidence suggests that combining structural and functional imaging data with circulating biomarkers may substantially improve risk stratification compared with either approach alone. Biomarkers reflecting myocardial injury, inflammation, thrombosis, lipid metabolism, and haemodynamic stress provide complementary information that may enhance individualized therapeutic decision-making [78].

Among thrombotic biomarkers, D-dimer remains one of the most widely used markers in clinical practice. Recent advances have focused on improving diagnostic specificity through age-adjusted cut-off strategies. Age-adjusted D-dimer thresholds have demonstrated improved diagnostic performance for excluding venous thromboembolism while reducing unnecessary imaging investigations in older patients [79]. Such approaches illustrate how laboratory biomarkers and imaging pathways can be integrated to optimize diagnostic efficiency and resource utilization.

The relationship between lipid metabolism and cardiovascular risk has also received considerable attention. While low-density lipoprotein cholesterol (LDL-C) remains a major therapeutic target for atherosclerotic cardiovascular disease prevention, recent evidence suggests that extremely low LDL-C levels may influence haemostatic balance and bleeding risk. In a large multinational analysis from the RIETE Registry, low LDL-C concentrations were associated with an increased risk of major bleeding in patients receiving anticoagulant therapy for venous thromboembolism [80]. These findings underscore the importance of considering competing thrombotic and haemorrhagic risks when developing personalized treatment strategies.

Similarly, growing evidence suggests that statins exert pleiotropic effects extending beyond lipid lowering. Experimental and clinical studies have demonstrated anti-inflammatory, endothelial, antithrombotic, and plaque-stabilizing properties that may contribute to improved cardiovascular outcomes [81]. A comprehensive review of the effects of statins on coagulation pathways highlighted multiple mechanisms through which these agents may influence thrombotic risk, including modulation of tissue factor expression, platelet activation, thrombin generation, and fibrinolytic activity [82].

Observational studies from large international registries have further suggested potential clinical benefits of statin therapy in patients with venous thromboembolism. Analyses from the RIETE Registry reported associations between statin use and lower mortality among patients with pulmonary embolism and deep vein thrombosis receiving anticoagulant therapy [83,84]. Although causal relationships cannot be established from observational data alone, these findings support ongoing interest in the role of adjunctive therapies within personalized antithrombotic management strategies.

The integration of imaging biomarkers with laboratory data is particularly relevant in patients with thromboembolic disease. Advanced imaging techniques allow assessment of right ventricular dysfunction, residual thrombotic burden, vascular remodeling, myocardial injury, and haemodynamic consequences of pulmonary embolism. When combined with biomarkers such as D-dimer, cardiac troponins, natriuretic peptides, inflammatory markers, and lipid parameters, these imaging findings may facilitate more accurate prediction of recurrence, bleeding complications, and long-term outcomes [85].

Artificial intelligence is expected to further enhance this multimodal approach. Future predictive models will likely integrate imaging features, radiomic signatures, wearable device data, laboratory biomarkers, genetic information, and electronic health records into comprehensive individualized risk profiles [86]. Such models may assist clinicians in selecting optimal diagnostic strategies, tailoring therapeutic interventions, and determining appropriate follow-up intensity.

Despite the considerable promise of AI-driven precision medicine, important challenges remain. Issues related to data quality, interoperability, algorithm transparency, ethical considerations, and external validation continue to limit widespread clinical implementation [87]. Furthermore, robust prospective studies are required to demonstrate that AI-supported decision-making translates into meaningful improvements in patient outcomes.

Nevertheless, the convergence of advanced imaging, artificial intelligence, digital health technologies, and biomarker-based risk assessment represents one of the most important developments in contemporary cardiovascular medicine. Rather than viewing imaging as an isolated diagnostic tool, future healthcare systems are likely to incorporate imaging findings into integrated predictive frameworks capable of supporting highly personalized management strategies.

**Figure 1:**
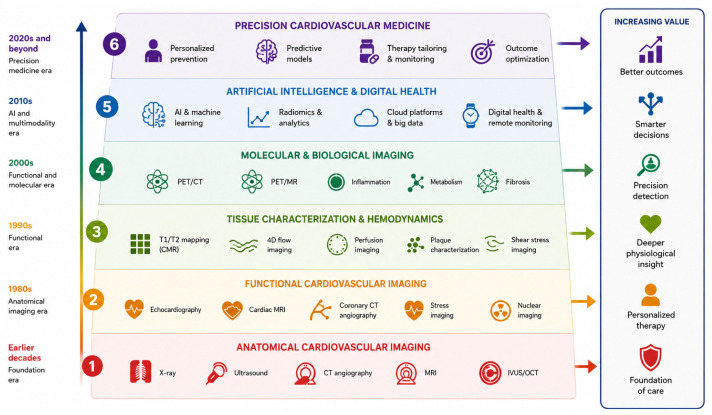
Evolution of cardiovascular imaging toward precision medicine. Legend: Schematic representation of the progressive evolution of cardiovascular imaging from anatomical assessment to functional evaluation, tissue characterization, molecular imaging, radiomics, artificial intelligence integration, and personalized cardiovascular medicine.

**Figure 2:**
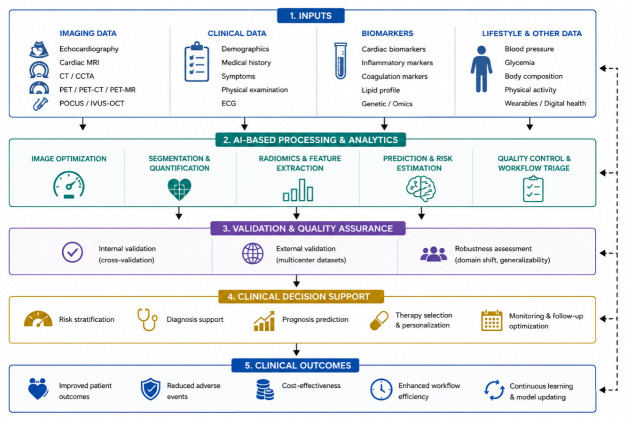
Integrated multimodal framework for personalized cardiovascular risk stratification. Legend: Conceptual model illustrating the interaction among advanced imaging modalities, laboratory biomarkers, artificial intelligence algorithms, digital health technologies, and clinical variables in supporting individualized cardiovascular diagnosis, prognosis, and therapeutic decision-making.

The concepts discussed throughout this review are summarized in Figure 1, which illustrates the evolution of cardiovascular imaging from anatomical assessment toward precision medicine. Furthermore, Figure 2 presents an integrated framework combining imaging biomarkers, laboratory parameters, artificial intelligence, and digital health technologies for personalized cardiovascular risk stratification. The principal interactions among these domains are also summarized in Table 2.

Overall, the future of cardiovascular imaging will not be defined solely by improvements in image quality or technological sophistication. Instead, its greatest impact will likely arise from the ability to integrate imaging with biological, clinical, and digital information, thereby transforming large volumes of data into actionable knowledge that improves patient outcomes and advances precision cardiovascular medicine.

## 9. Discussion

Cardiovascular imaging is currently undergoing one of the most profound transformations in its history. What was once primarily a discipline focused on anatomical visualization has evolved into a multidimensional platform capable of characterizing myocardial structure, tissue composition, vascular biology, haemodynamics, inflammation, metabolism, and disease activity. This transition has significantly expanded the clinical value of imaging and has established advanced imaging modalities as essential components of contemporary cardiovascular medicine.

The technologies discussed in this review highlight the remarkable breadth of current innovation. Advanced echocardiographic techniques, including strain imaging, myocardial work analysis, contrast-enhanced stress echocardiography, and novel approaches to myocardial scar detection, have substantially improved functional assessment and prognostic stratification. Cardiovascular magnetic resonance has emerged as the reference standard for tissue characterization, providing detailed insights into fibrosis, inflammation, myocardial viability, and vascular haemodynamics. At the same time, computed tomography has evolved beyond anatomical coronary assessment toward characterization of plaque vulnerability, vascular inflammation, and biological disease activity, with photon-counting CT representing one of the most promising technological advances in the field.

Molecular imaging modalities such as PET/CT and PET/MR have further expanded diagnostic capabilities by enabling visualization of metabolic and inflammatory processes that underlie cardiovascular disease progression. Similarly, intravascular imaging techniques including IVUS and OCT have enhanced understanding of plaque morphology and have improved the precision of coronary interventions. The growing adoption of point-of-care ultrasound has also extended the benefits of cardiovascular imaging beyond specialized centres, facilitating rapid bedside assessment and improving access to diagnostic information across diverse healthcare settings.

Importantly, the future impact of cardiovascular imaging will depend not only on technological improvements but also on the ability to integrate imaging findings with complementary sources of information. Artificial intelligence, machine learning, radiomics, digital health technologies, and biomarker-based risk assessment are increasingly converging with advanced imaging to create comprehensive precision medicine frameworks. These approaches have the potential to move clinical practice beyond population-based treatment strategies and toward truly individualized cardiovascular care.

The integration of imaging biomarkers with laboratory parameters, clinical characteristics, wearable-device data, and predictive algorithms may improve diagnostic accuracy, refine risk stratification, and support more personalized therapeutic decisions. Such multimodal strategies appear particularly relevant in complex conditions characterized by heterogeneous clinical presentations and variable outcomes, including coronary artery disease, heart failure, pulmonary embolism, venous thromboembolism, and arrhythmic disorders.

Despite these advances, several challenges remain. Standardization of acquisition protocols, validation of emerging biomarkers, interoperability of digital platforms, regulatory considerations, and equitable access to advanced technologies represent important priorities for future research. Furthermore, robust prospective studies are needed to determine whether imaging-guided and AI-assisted management strategies consistently translate into improved clinical outcomes and cost-effectiveness.

Despite the remarkable progress achieved by AI, important barriers remain before widespread clinical implementation can be achieved. A major challenge is domain shift, whereby algorithm performance may deteriorate across different scanner vendors, acquisition protocols, institutions, and patient populations. Consequently, AI systems require rigorous external validation using large multicentre datasets representative of real-world clinical practice. Prospective clinical studies evaluating patient-centred outcomes, workflow efficiency, diagnostic accuracy, and cost-effectiveness remain limited. In addition, regulatory approval, algorithm transparency, explainability, cybersecurity, and continuous post-market monitoring will be essential to ensure safe and reliable implementation of AI-assisted cardiovascular imaging in routine clinical care.

One of the major barriers to clinical implementation is domain shift, whereby algorithm performance may deteriorate when applied across different scanner vendors, acquisition protocols, institutions, and patient populations. Consequently, AI algorithms require rigorous multicentre external validation before routine implementation. Furthermore, prospective studies evaluating patient-centred outcomes, workflow efficiency, cost-effectiveness, and regulatory compliance remain essential to ensure safe clinical deployment.

### Current Limitations

Despite remarkable technological advances, widespread implementation of next-generation cardiovascular imaging remains limited by high costs, limited availability of advanced imaging systems, operator dependency, variability among vendors and acquisition protocols, insufficient external validation of AI algorithms, and the need for dedicated multidisciplinary expertise. Future research should therefore focus not only on technological innovation but also on standardization, cost-effectiveness, multicentre validation, and integration of advanced imaging into sustainable clinical workflows.

## 10. Future Perspectives

Future progress in cardiovascular risk stratification will likely depend less on the introduction of entirely new imaging modalities than on the intelligent integration of existing technologies. Rather than considering imaging techniques as isolated diagnostic tools, future clinical pathways should combine low-cost, widely available tests—including digital ECG, point-of-care ultrasound, biomarkers, arterial stiffness assessment, and AI-assisted clinical prediction—with advanced imaging reserved for selected high-risk patients. Artificial intelligence will probably play a central role by integrating multimodal data into individualized risk prediction models capable of improving diagnostic accuracy, optimizing healthcare resources, and supporting personalized preventive strategies. Such an approach may increase diagnostic performance while maintaining sustainability within real-world healthcare systems.

## 11. Conclusions

In conclusion, cardiovascular imaging is rapidly evolving from a diagnostic discipline into a central pillar of precision cardiovascular medicine. The integration of advanced imaging technologies with artificial intelligence, molecular biomarkers, and digital health solutions offers unprecedented opportunities to improve disease characterization, personalize treatment strategies, and enhance patient outcomes. As these innovations continue to mature, the next generation of cardiovascular imaging is likely to play an increasingly important role in bridging the gap between technological progress and individualized patient care.

## Figures and Tables

**Table 1:** Current and Emerging Cardiovascular Imaging Modalities: Clinical Applications, Advantages, Limitations, and Future Perspectives.

Imaging Modality	Main Clinical Applications	Major Advantages	Principal Limitations	Future Perspectives
Echocardiography	Cardiac structure and function assessment, valvular disease, heart failure	Widely available, portable, radiation-free, low cost	Operator dependency, acoustic window limitations	AI-assisted acquisition and interpretation, advanced strain analysis
Stress Echocardiography	Ischemia detection, coronary microvascular dysfunction, risk stratification	Functional assessment, no radiation	Image quality dependence	Automated perfusion and myocardial work analysis
Cardiovascular Magnetic Resonance (CMR)	Tissue characterization, fibrosis, myocarditis, cardiomyopathies	Excellent tissue characterization, no ionizing radiation	Cost, availability, contraindications in selected patients	Quantitative mapping, 4D-flow imaging, AI-assisted analysis
Coronary CT Angiography (CCTA)	Coronary artery disease diagnosis, plaque characterization	High diagnostic accuracy, non-invasive coronary imaging	Radiation exposure, contrast administration	Advanced plaque phenotyping, radiomics
Photon-Counting CT (PCCT)	Coronary plaque assessment, vascular imaging	Higher spatial resolution, lower noise, improved tissue characterization	Limited availability, high costs	Precision plaque biology and molecular characterization
PET/CT	Vascular inflammation, myocardial viability, infection imaging	Molecular imaging capabilities	Radiation exposure, limited availability	Novel tracers targeting inflammation and plaque activity
PET/MR	Inflammation, metabolism, infiltrative cardiomyopathies	Combined molecular and tissue characterization	High cost, technical complexity	Integrated precision imaging platforms
IVUS	Plaque burden assessment, PCI guidance	Deep vessel wall visualization	Invasive procedure	Hybrid imaging systems and AI-assisted analysis
OCT	Plaque microstructure characterization, PCI optimization	Very high spatial resolution	Limited penetration depth	Automated plaque phenotyping
Point-of-Care Ultrasound (POCUS)	Bedside cardiovascular and vascular assessment	Portable, rapid, accessible	Operator dependency, limited comprehensive evaluation	Handheld AI-enabled devices and tele-ultrasound

**Table 2:** Integration of Imaging Biomarkers, Artificial Intelligence, and Clinical Variables for Personalized Cardiovascular Risk Stratification.

Clinical Scenario	Imaging Biomarkers	Laboratory Biomarkers	AI/Digital Health Applications	Potential Clinical Impact
Coronary Artery Disease	Plaque burden, high-risk plaque features, myocardial ischemia	LDL-C, hs-CRP, troponins	Event prediction models	Personalized preventive strategies
Heart Failure	GLS, myocardial fibrosis, ventricular remodeling	NT-proBNP, troponin	Remote monitoring and outcome prediction	Optimized therapy selection
Arrhythmic Risk	Myocardial scar burden, ventricular dysfunction	Troponin, inflammatory markers	Sudden death prediction models	Improved ICD candidate selection
Pulmonary Embolism	Right ventricular dysfunction, clot burden	D-dimer, troponin, BNP	Automated risk classification	Tailored treatment intensity
Venous Thromboembolism	Residual thrombosis, venous ultrasound findings	D-dimer, lipid profile	Recurrence prediction algorithms	Individualized anticoagulation strategies
Bleeding Risk During Anticoagulation	Vascular imaging markers, frailty assessment	LDL-C, hemoglobin, platelet count	Integrated bleeding-risk models	Safer antithrombotic management
Acute Cardiac Events	Multimodal imaging findings	Biomarker panels	Mobile health alert systems	Earlier diagnosis and intervention
Population Screening	CAC score, POCUS findings	Traditional risk factors	AI-enhanced screening pathways	Precision prevention strategies

**Table 3:** Representative Artificial Intelligence Applications Across Cardiovascular Imaging Modalities.

Modality	AI Application	Clinical Task	Level of Clinical Maturity
Echo	Automated acquisition	Routine	Clinical
Echo	GLS segmentation	Clinical	Clinical
CMR	LV segmentation	Clinical	Clinical
CMR	Scar quantification	Clinical	Emerging
CT	Plaque analysis	Clinical	Clinical
CT	Radiomics	Research	Emerging
PET	Quantitative analysis	Research	Emerging
IVUS/OCT	Plaque phenotyping	Emerging	Research

Legend: The table summarizes current AI-assisted applications throughout the cardiovascular imaging workflow, including image acquisition, reconstruction, segmentation, quantitative analysis, radiomics, and clinical decision support, together with their principal clinical applications and current level of implementation.

## Data Availability

No new data were created or analyzed in this study. Data sharing is not applicable to this article.
